# Genotype-dependent stability and specialization of arbuscular mycorrhizal fungal communities under drought in common bean

**DOI:** 10.3389/fpls.2026.1786322

**Published:** 2026-05-01

**Authors:** Maycon Cristiano Barbosa, Thierry Alexandre Pellegrinetti, Izadora de Cássia Mesquita da Cunha, Ana Vitória Reina da Silva, Eduardo Henrique Marcandalli Boleta, Lara de Almeida Losovoi, Rodrigo Mendes, Siu Mui Tsai, Lucas William Mendes

**Affiliations:** 1 Center for Nuclear Energy in Agriculture, University of São Paulo, Piracicaba, SP, Brazil; 2 Brazilian Agricultural Research Corporation, Embrapa Environment, Jaguariuna, Brazil

**Keywords:** climate change, ITS, microbial ecology, phaseolus vulgaris, plant-microbe interaction

## Abstract

Arbuscular mycorrhizal fungi (AMF) represent a key biological strategy for enhancing agricultural resilience under extreme climatic events such as drought. However, how AMF interact with drought-tolerant plant genotypes to sustain performance under water limitation remains poorly understood. Here, we used high-throughput DNA sequencing to investigate AMF communities associated with drought-tolerant (BAT477 and SEA5) and drought-susceptible (IAC-Milênio and IAC-80SH) common bean genotypes, integrating taxonomic, structural, and functional perspectives under contrasting water regimes. We hypothesized that drought tolerance is not simply linked to AMF presence, but rather to the ability of host genotypes to structure and stabilize their mycorrhizal communities under stress. Our results reveal genotype-specific responses to drought, with distinct community restructuring dynamics observed across individual genotypes. Drought-tolerant genotypes maintained or increased AMF relative abundance, diversity, and functional integrity under drought, whereas susceptible genotypes displayed opposing changes in the community's structure. Although most genotypes displayed high dissimilarity in AMF community structure between control and drought conditions, tolerant genotypes reorganized their communities through increase in the relative abundance of key ASVs, whereas susceptible genotypes experienced substantial reductions in abundance, diversity, and specialist ASVs. Niche occupancy and functional guild analyses further showed that AMF communities in tolerant genotypes were dominated by specialist and symbiotrophic ASVs, whereas susceptible genotypes shifted toward rare and functionally reduced assemblages. At the plant level, AMF community stability was positively associated with root biomass and negatively associated with foliar nutrient, indicating a tight coupling between mycorrhizal community structure, host nutritional status, and growth. Collectively, our findings indicate that drought tolerance in common bean emerges, at least in part, from a cooperative host–microbiome strategy in which the host actively regulates the structure and functional stability of AMF communities under water stress. These results advance our understanding of plant-mycorrhizal interactions in drought adaptation and highlight the potential of integrating mycorrhizal functionality into plant breeding strategies aimed at developing climate-resilient crops.

## 1 Introduction

Drought is one of the most severe consequences of global climate change, significantly impairing agricultural productivity through both direct effects on plant physiology and indirect effects on soil biological processes (Seleiman et al., 2021). In response, the development of drought-tolerant cultivars through plant breeding or genetic engineering has become a central strategy for mitigating the impacts of water scarcity (Cooper and Messina, 2022; Guadarrama-Escobar et al., 2024). Recent evidence further indicates that host-driven selection of the soil microbiome plays an active role in modulating plant responses to drought, positioning belowground microbial communities as functional drivers of stress resilience rather than passive system components (Kumar and Verma, 2018; Mendes et al., 2017; Silva et al., 2025; Singh and Singh, 2024). Despite these advances, microbiome-informed traits and plant–microbe compatibility are still rarely incorporated into conventional plant breeding programs, representing a significant bottleneck for the development of resilient cropping systems.

Soil microbes can act as belowground defenders by enhancing soil structure, nutrient acquisition, and plant performance under water-limited conditions (Araujo et al., 2025a). Among them, arbuscular mycorrhizal fungi (AMF) play a pivotal role in promoting plant growth and enhancing tolerance to abiotic and biotic stresses. These obligate biotrophs establish symbiotic associations with plant roots through finely regulated molecular signaling pathways, including the perception of host-derived strigolactones (Augé, 2001; Bednarek et al., 2010; Haichar et al., 2014; Xu et al., 2023; Araujo et al., 2025b). AMF symbiosis contributes to the maintenance of plant water status by improving hydraulic conductivity, regulating stomatal behavior, enhancing antioxidant defenses, and modulating the expression of mycorrhiza-associated aquaporin genes (Das and Sarkar, 2024; Yang et al., 2025). However, the contribution of AMF to drought tolerance cannot be fully understood without considering their ecological organization at the community level.

Importantly, the ecological functioning of arbuscular mycorrhizal fungi is not determined solely by their presence or colonization levels, but also by the structure, diversity, and stability of AMF communities in the rhizosphere (Powell and Rillig, 2018). Community-level attributes, such as taxonomic composition, dominance patterns, and specialization, can strongly influence nutrient exchange efficiency, stress buffering capacity, and the resilience of plant-fungal symbioses under environmental perturbations (Chagnon et al., 2013). However, how drought reshapes AMF community structure in a genotype-dependent manner, and whether tolerant genotypes are able to maintain more stable or functionally specialized AMF assemblages under water deficit, remains poorly understood.

A recent study showed that drought-tolerant common bean genotypes (BAT477 and SEA5) exhibit enhanced physiological performance and recruit rhizosphere microbiomes enriched in functional genes associated with osmoprotection, nutrient cycling, and stress adaptation, whereas susceptible cultivars display more reactive microbial functional profiles (Silva et al., 2025). These findings highlighted the importance of plant–microbiome interactions in drought resilience and emphasized the need to move beyond functional potential toward the identification of key microbial taxa and community configurations underlying stress tolerance. However, how these host-driven microbial shifts translate into changes in the composition and ecological structure of arbuscular mycorrhizal fungal communities remains poorly understood.

This knowledge gap is particularly relevant for common bean cultivation (*Phaseolus vulgaris* L.) in Brazil, one of the world’s largest producers and consumers of common beans, with annual production ranging from 2.7 to 3.4 million tons and a cultivated area of approximately 2.9 million hectares (Clemente et al., 2017; Mantovani et al., 2024). In addition, drought and irregular rainfall remain the most significant constraints on common bean production, especially in rainfed systems, which account for more than 90% of the cultivated area in Brazil and are associated with heightened production risks (Heinemann et al., 2016; Justino et al., 2025; Massignam et al., 2017).

Building on advances in next-generation sequencing approaches and the urgent need to improve drought resilience in common bean production systems, this study aimed to taxonomically characterize and evaluate the community structure of AMF assemblages in the rhizosphere of common bean genotypes contrasting in drought tolerance. We hypothesized that drought tolerance in common bean is partly mediated by genotype-specific symbiotic associations with AMF communities, enabling tolerant cultivars to maintain more stable and specialized fungal assemblages under water-deficit conditions.

## 2 Materials and methods

### 2.1 Experimental design

Soil and rhizosphere samples used in this study were obtained from a mesocosm experiment conducted at the Luiz de Queiroz College of Agriculture, University of São Paulo, in Piracicaba, Brazil (Silva et al., 2025). The experiment was established in polyethylene pots (30 cm height × 20 cm diameter), each filled with approximately 6 kg of dry, medium-textured Red–Yellow Latosol, and maintained under controlled greenhouse conditions in a randomized complete block design with five replicates per treatment. The experimental design followed a factorial arrangement including four common bean (*P. vulgaris* L.) cultivars contrasting in drought tolerance, a plant-free bulk soil control (basal levels of control without root influence), and two water regimes (well-watered and drought stress), resulting in a total of 50 experimental units [(four genotypes + one bulk soil) × two water regimes × five replicates]. The drought-tolerant genotypes SEA5 (Briñez et al., 2017) and BAT477 (Recchia et al., 2018), as well as the drought-susceptible genotypes IAC-Carioca 80SH (Blair et al., 2010) and IAC-Milênio (Carbonell et al., 2014), were previously characterized based on their physiological performance and yield under water-deficit conditions (Asfaw and Blair, 2011; Polania et al., 2017; Recchia et al., 2018).

Plants were initially grown under uniform irrigation at 85% water-holding capacity (WHC) until the pre-flowering developmental stage (R5; approximately 50 days after sowing), when the plants were submitted to a pre-drought for three days. Drought stress was then applied by reducing soil moisture to 40% WHC over 96 h, while well-watered control plants were maintained at 85% WHC (Recchia et al., 2018). Bulk soil and rhizosphere samples were collected after 96 h of drought stress. Bulk soil samples were obtained from plant-free pots used as controls, whereas rhizosphere soil was collected by gently shaking the roots to retain soil adhering to the root surface. All samples were stored at −20 °C until further processing.

Plant biometric, physiological, nutritional, and soil chemical parameters were obtained from the same mesocosm experiment described by Silva et al. (2025). Briefly, plant height and gas exchange parameters were measured, including photosynthetic rate, stomatal conductance, intercellular CO_2_ concentration, and transpiration, using an infrared gas analyzer (IRGA; LI-COR XT 6400). For soil chemical characterization, composite soil samples were collected and analyzed following standard procedures described by Camargo et al. (2009). Plant shoots and roots were harvested separately to determine fresh and dry biomass after oven drying at 65 °C for 72 h. Foliar nutrient concentrations (P, K, Ca, Mg, S, B, Cu, Fe, Mn, and Zn) were quantified by ICP-MS, while total nitrogen was determined by sulfuric acid digestion followed by Kjeldahl distillation, according to Malavolta et al. (1989).

### 2.2 DNA extraction, sequencing, and data processing

Soil DNA was extracted from the 50 samples using the DNeasy PowerSoil^®^ Kit (QIAGEN), following the manufacturer’s instructions. The fungal ITS region was amplified using the primers ITS5-1737F (5′-GGAAGTAAAAGTCGTAACAAGG-3′) and ITS2-2043R (5′-GCTGCGTTCTTCATCGATGC-3′), generating amplicons of approximately 200 bp–400 bp. Amplicon library preparation and sequencing were performed by Novogene Co. Ltd. (Sacramento, CA, USA), following the company’s standard protocols for fungal ITS sequencing on the Illumina NovaSeq platform (Illumina Inc., San Diego, CA, USA) using paired-end 2 × 250 bp reads, with an average output of approximately 100,000 reads per sample.

Sequence data were processed using QIIME2 version 2024.5.0 (Bolyen et al., 2019). Following demultiplexing, quality filtering and denoising were performed with the DADA2 plugin (Callahan et al., 2017), using the consensus approach for error correction and the identification and removal of chimeric sequences. Low-quality reads (Phred score <20) were excluded, and singleton and doubleton ASVs were removed after denoising to reduce sequencing noise. After quality control, a total of 7,759,190 high-quality sequences were retained, with an average of 155,183 reads per sample. To account for differences in sequencing depth, samples were rarefied to 89,600 sequences, corresponding to the lowest read count among samples. Taxonomic assignment was performed using the UNITE database version 9.0 (Nilsson et al., 2019). The resulting ASV tables were used to assess AMF community composition, structure, and diversity through bioinformatic and multivariate statistical analyses. All downstream analyses were conducted by comparing drought and well-watered conditions within each common bean genotype. The sequences are publicly available at NCBI SRA under the accession PRJNA1392414.

### 2.3 Data analysis

Beta diversity was assessed using Principal Coordinates Analysis (PCoA) based on Bray–Curtis dissimilarity to reduce dimensionality and visualize AMF group separation between water regimes and soil chemical parameters for each bean genotype, with data log-transformed as log(1 + x). Statistical significance was tested using multivariate analysis of variance (MANOVA, p <0.05). Alpha diversity was assessed using ASV richness (S) and Shannon’s diversity index (H’), and was compared using the Kruskal–Wallis test (p <0.05) without multiple-testing correction, implemented in the vegan package (Oksanen, 2012; Oksanen et al., 2025). S was defined as the total number of AMF ASVs per sample. The H’ diversity index was calculated as H = −Σ (Xi/Xo) × log(Xi/Xo), where Xi represents the number of sequences of ASV i and Xo corresponds to the total number of sequences of all ASVs within a sample (Shannon, 1948). Taxonomic composition at the family and genus levels was visualized using the Microeco package (Liu et al., 2021), and ASV sequence-level sharing among genotypes was represented using Venn diagrams (Chen and Boutros, 2011).

AMF community dissimilarity was further evaluated using Similarity Percentage Analysis (SIMPER) (Clarke, 1993), implemented with the vegan package. SIMPER partitions Bray–Curtis dissimilarity between pairs of environmental contrasts to determine the individual contribution of each ASV to overall differences. Statistical significance was tested using 999 permutations, and ASVs with p ≤0.05 were considered significant contributors. ASV abundance matrices for the phylum Glomeromycota were analyzed using the interaction term “Cultivar × Stress.” Results were summarized up to 90% cumulative contribution, and the 20 most influential ASVs per comparison were extracted. Mean abundance was used to construct bubble plots and cumulative contribution curves, allowing visualization and comparison of the relative impact of ASVs between contrasts for each cultivar.

To validate AMF community responses, we applied the multinomial species classification method (CLAM test) described by Chazdon et al. (2011) to assess the niche occupancy patterns of ASV taxa under contrasting water regimes. Analyses were conducted separately for each genotype by comparing samples under control and drought conditions. The test classifies ASVs into four categories based on their abundance distribution across the two conditions: (i) specialists of the control condition, (ii) specialists of drought, (iii) generalists (similarly distributed between conditions), and (iv) too rare to classify. Taxonomic classification was based on abundance counts, using a significance level of p = 0.05 and the default specialization threshold (K = 2/3), meaning that taxa with at least two-thirds of their total abundance associated with one condition were classified as specialists of that condition. Taxa with insufficient abundance for reliable classification were assigned to the “too rare” category.

Furthermore, taxonomic data were mapped to symbiotic trophic modes using the FUNGuild database (Nguyen et al., 2016) in the Python language. FUNGuild is a two-component system composed of a taxon parser and a guild parser that queries the FUNGuild database to assign ecological functions to ASVs. Guild assignment followed the convention of conserving genus-level limits for many fungal taxa and retaining sequences with >93% similarity to reference sequences. Guild-level symbiotrophs were compared using PERMANOVA (p <0.05), and the arbuscular mycorrhizal guild was compared between environmental contrasts within each bean genotype using the Wilcoxon test (p <0.05).

Finally, correlations between plant physiological and nutritional traits and AMF were assessed using the Microeco package (Liu et al., 2021). Compositional dissimilarity was estimated using Bray–Curtis distance, and pairwise correlations between each plant variable and the relative abundance of Glomeromycota ASVs were computed using Spearman’s coefficient with Benjamini–Hochberg false discovery rate (FDR) correction (p <0.05). For physiological, nutritional, and morphometric variables showing significant correlations with AMF community structure, correlation models were fitted between environmental variables and Bray–Curtis distances, enabling visualization of the direction and intensity of plant–AMF associations. The average data tables used for correlation models are available in the **Supplementary Material** (**Supplementary Tables 1**, **2**).

Here, we adopted statistical approaches tailored for microbiome datasets characterized by high sparsity and skewed distributions, and appropriate to the size of our dataset. PCoA is widely used to reduce data dimensionality and to represent differences among samples using distinct distance matrices (Khomich et al., 2021; Paliy and Shankar, 2016; Wang et al., 2022). The log1p transformation, in turn, is useful for controlling highly skewed microbial distributions and extreme values, improving the performance of statistical tests while avoiding issues associated with undefined log(0) values in sparse datasets (West, 2022). The use of multiple-testing corrections in sparse datasets can markedly reduce statistical power and hinder the detection of true positives (Buttigieg and Ramette, 2014; Hawinkel et al., 2019; Pascovici et al., 2016). Finally, all diversity, SIMPER, CLAM, and correlation analyses were performed at the ASV (amplicon sequence variant) level without prior taxonomic aggregation. Taxonomic assignments were used exclusively for descriptive visualization at the family and genus levels.

## 3 Results

### 3.1 AMF community structure in the rhizosphere under drought-stress

**Figure 1:**
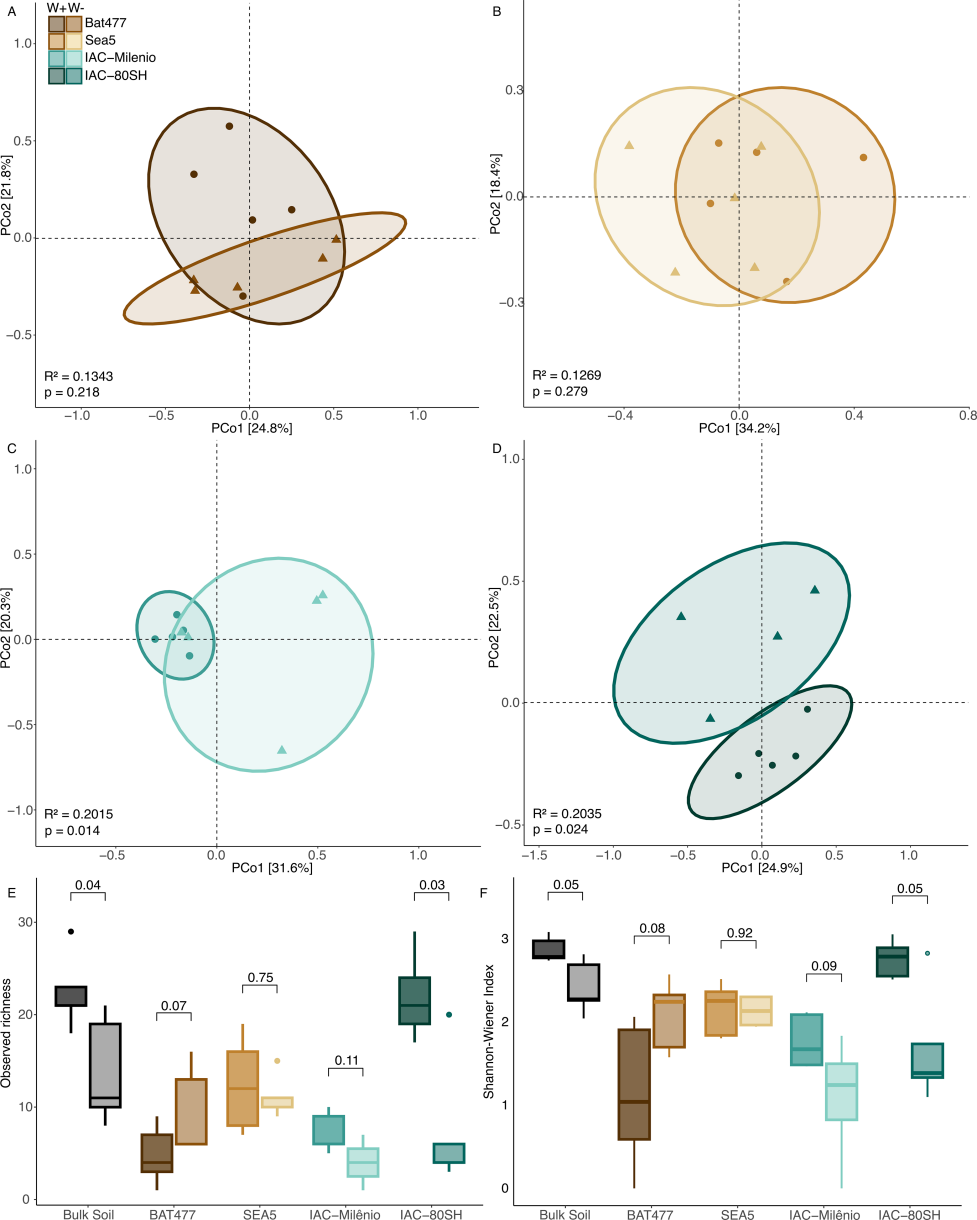
Effect of drought on the structure and diversity of arbuscular mycorrhizal fungal (AMF) communities in the rhizosphere of four common bean cultivars. Principal Coordinate Analysis (PCoA) of AMF community composition for drought-tolerant cultivars **(A)** BAT477 and **(B)** SEA5, and drought-susceptible cultivars **(C)** IAC-Milênio and **(D)** IAC-80SH. The analysis considered only the drought event as the explanatory variable, and MANOVA statistics are shown in the lower right corner of each plot. **(E)** ASV richness and **(F)** diversity (Shannon–Wiener index) were assessed at the ASV level; error bars represent the mean of five biological replicates, and numerical values correspond to Kruskal–Wallis test results (p <0.05). All plots were generated in R.

We found that arbuscular mycorrhizal fungi (AMF) community structure changed in the rhizosphere in response to drought depending on the common bean genotype (**Figure 1**). In the drought-tolerant cultivars BAT477 and SEA5, AMF community structure did not differ significantly between well-watered and drought conditions (MANOVA BAT477: R^2^ = 0.1343, p = 0.218; MANOVA SEA5: R^2^ = 0.1269, p = 0.279), indicating a stable community configuration under water deficit (**Figures 1A, B**). In contrast, drought significantly altered the AMF community structure in the rhizosphere of the drought-susceptible cultivars IAC-Milênio and IAC-80SH (MANOVA IAC-Milênio: R^2^ = 0.2015, p = 0.014; MANOVA IAC-80SH: R^2^ = 0.2035, p = 0.024), revealing a pronounced restructuring of AMF assemblages under water stress (**Figures 1C, D**). No significant association was detected between AMF community structure and measured soil chemical variables within the experimental system (**Supplementary Tables 3**, **4**, **Supplementary Figure 1**).

Patterns of alpha diversity further supported these contrasting responses. We found that AMF richness and diversity differed significantly between drought and control conditions only in BAT477 and IAC-80SH (p <0.05). Notably, BAT477 exhibited an increase in both observed richness and diversity under drought, whereas IAC-80SH showed a marked reduction in these metrics (**Figures 1E, F**). In contrast, we detected no significant changes in richness or diversity for SEA5 or IAC-Milênio.

**Figure 2:**
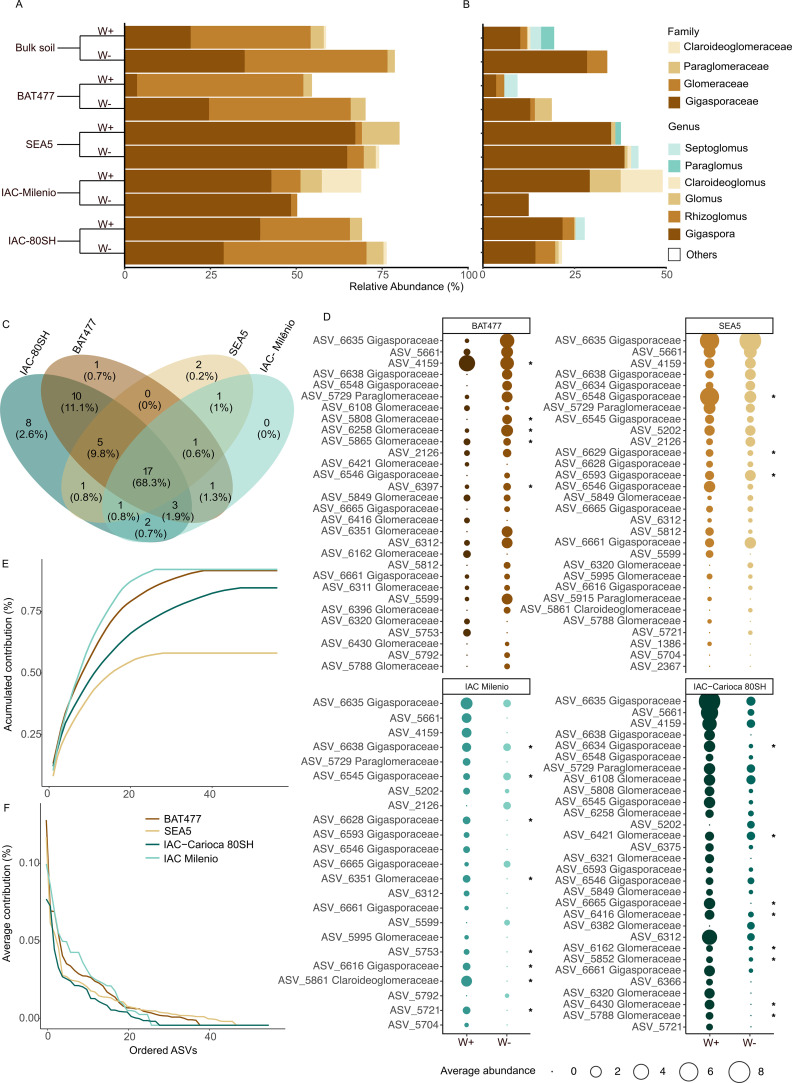
Composition and similarity of arbuscular mycorrhizal fungi (AMF) in the rhizosphere microbiome of drought-tolerant and drought-susceptible common bean cultivars. Relative abundance of AMF at the **(A)** family and **(B)** genus levels for bulk soil and all genotypes. **(C)** Venn diagram representing the shared ASV core microbiome. **(D)** Similarity Percentage Analysis (SIMPER) was performed using 999 permutations and a 90% cumulative contribution threshold. Bubble plots represent AMF communities in control and drought contrasts for each cultivar. **(E)** Line plots show the cumulative contribution of each ASV to Bray–Curtis dissimilarity between environmental contrasts for each cultivar, and **(F)** the mean contribution of each ASV to Bray–Curtis dissimilarity across contrasts for each cultivar.

Across all samples, the AMF community was composed of four main families: Gigasporaceae, Glomeraceae, Paraglomeraceae, and Claroideoglomeraceae (**Figure 2A**). Gigasporaceae and Glomeraceae dominated the community, accounting for the highest relative abundances across genotypes and water regimes, whereas Paraglomeraceae and Claroideoglomeraceae occurred at lower abundances and were not consistently detected across all environmental contrasts. At the genus level, taxonomic assignment using the UNITE database allowed the classification of 23.3% of ASVs, corresponding to the genera *Gigaspora*, *Glomus*, *Rhizoglomus*, *Claroideoglomus*, *Septoglomus*, and *Paraglomus* (**Figure 2B**). Despite these differences, tolerant and susceptible cultivars shared a substantial core AMF assemblage, with 68% of ASVs common across genotypes (**Figure 2C**). It is important to note that only 23.3% of ASVs could be confidently assigned at the genus level. Therefore, interpretations regarding taxonomic identity are restricted to this subset, whereas structural inferences are based on full ASV-level resolution. It should also be noted that ITS-based metabarcoding may underestimate part of Glomeromycota diversity due to primer bias; however, this marker has been widely used to explore ecological patterns of AMF communities when interpreted cautiously (Berruti et al., 2017).

At the ASV level, approximately 20 ASVs accounted for most of the Bray–Curtis dissimilarity between environmental contrasts in all cultivars, with each of these ASVs contributing a non-zero fraction to community differentiation (**Figures 2D–F**). In the drought-tolerant genotypes BAT477 and SEA5, the top 20 ASVs explained approximately 70% and 50% of the dissimilarity between drought and well-watered conditions, respectively, while total dissimilarity reached 91.6% in BAT477 and 58.1% in SEA5 (**Figure 2E**). In contrast, for the drought-susceptible genotypes IAC-Milênio and IAC-80SH, the top 20 ASVs accounted for approximately 80% and 60% of the dissimilarity, respectively, with total dissimilarity values of 92.0% and 84.5% (**Figure 2E**).

Consistent with these patterns, tolerant cultivars displayed either maintenance (SEA5) or increases (BAT477) in the relative abundance of the top ASVs under drought, whereas susceptible cultivars exhibited marked reductions in the abundance of the top ASVs (**Figure 2D**). Accordingly, five and three ASVs showed significant abundance changes between environmental contrasts in BAT477 and SEA5, respectively (p <0.05), compared with eight significantly affected ASVs in each of the susceptible genotypes IAC-Milênio and IAC-80SH (p <0.05).

### 3.2 Maintenance of specialized AMF assemblages in the rhizosphere

**Figure 3:**
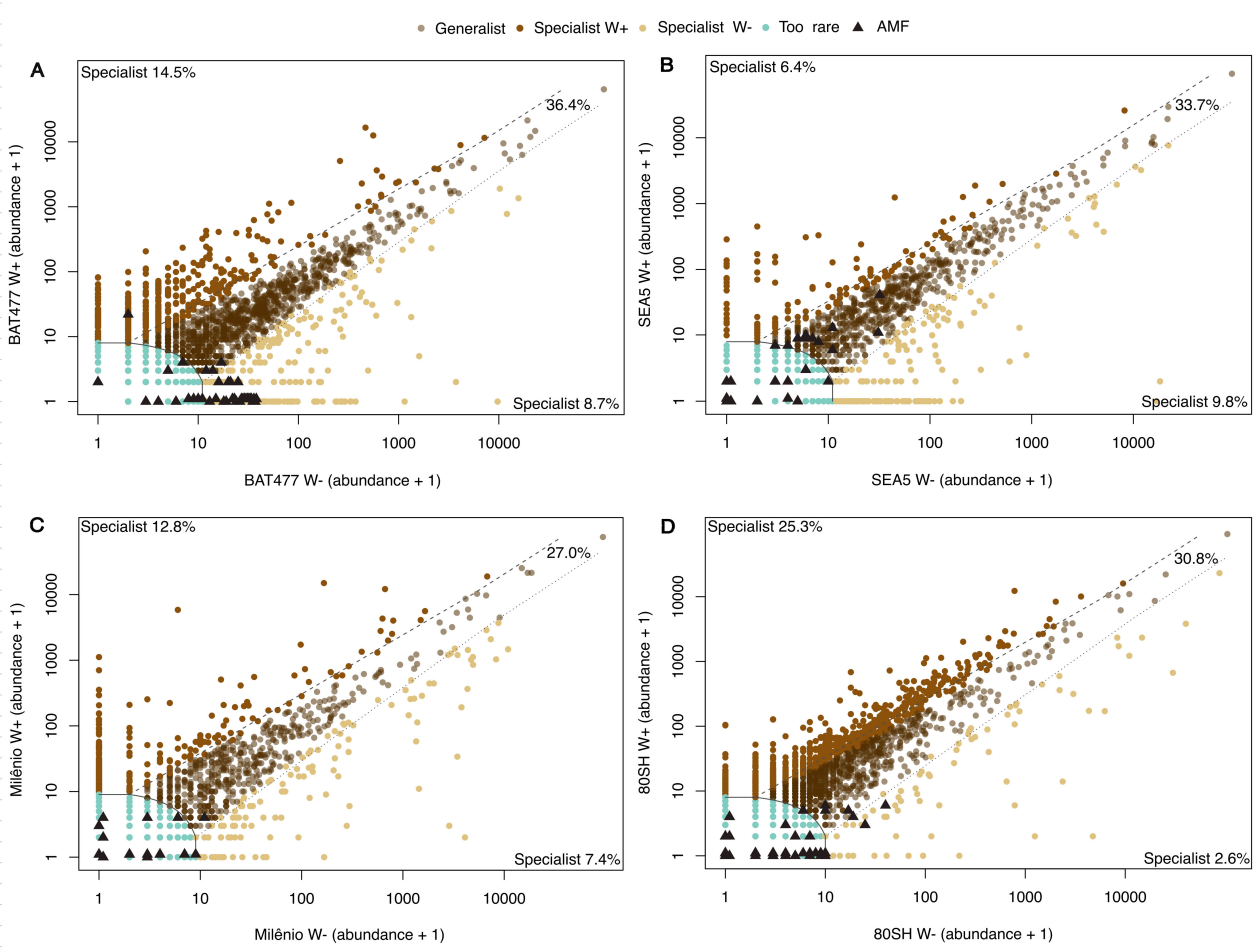
Niche occupancy classification of arbuscular mycorrhizal fungi (AMF) using the multinomial species classification method (CLAM). Pairwise comparisons were performed separately for each common bean genotype: **(A)** BAT477, **(B)** SEA5, **(C)** IAC-Milênio, and **(D)** IAC-80SH. ASVs were classified as habitat generalists (translucent brown), drought specialists (W−, yellow-brown), control specialists (W−, brown), or too rare for reliable classification (green), based on relative abundance and occurrence frequency between compartments. Black triangles indicate ASVs identified as AMF taxa. Proportions represent the fraction of ASVs assigned to each ecological category within each genotype.

Niche occupancy analysis showed that the relative contributions of generalist, specialist, and rare AMF ASVs varied among cultivars and between water regimes (**Figure 3**; **Supplementary Table 5**). Across all cultivars, generalist ASVs consistently represented the largest fraction of the AMF community, exceeding the proportion of specialist taxa under both well-watered and drought conditions. Among the drought-tolerant cultivars, contrasting patterns were observed. BAT477 showed a higher contribution of specialist AMF ASVs under drought conditions, indicating a shift toward niche specialization in response to water limitation (**Figure 3A**). In contrast, SEA5 exhibited a relative increase in generalist ASVs under drought, despite also showing a modest increase in specialist ASVs compared with the well-watered condition (**Figure 3B**). For the drought-susceptible cultivars, we observed a marked reduction in specialist ASVs under drought stress and a concomitant increase in the contribution of rare sequence variants. This pattern was particularly pronounced in IAC-80SH, which displayed a decline in specialist AMF under drought (**Figure 3D**), while IAC-Milênio also showed a clear shift toward niche occupancy dominated by rare ASVs (**Figure 3C**).

**Figure 4:**
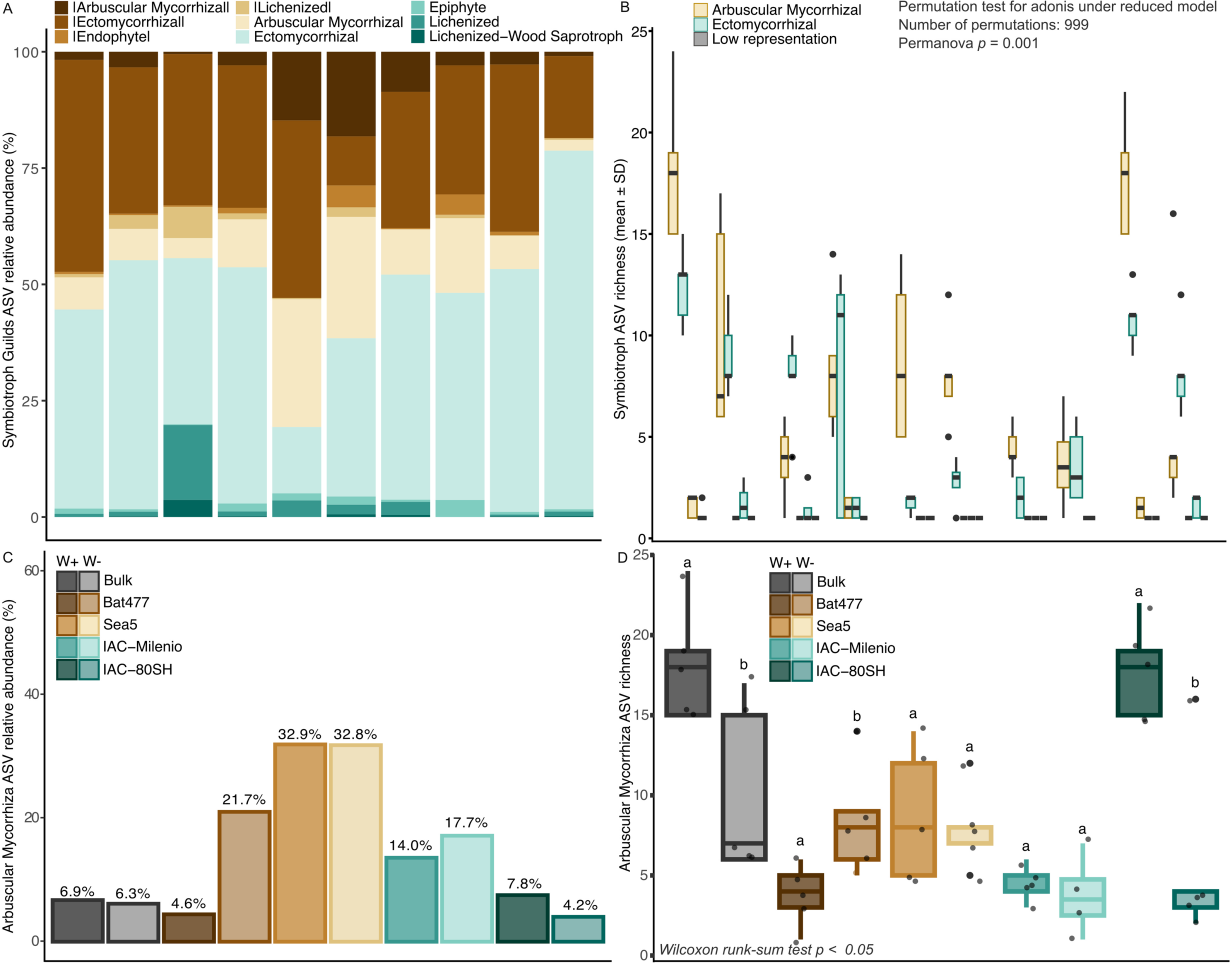
Functional prediction of fungal guilds under drought and control conditions in drought-tolerant and drought-susceptible common bean cultivars based on the FUNGuild database. **(A)** Relative abundance of all symbiotic fungal guilds and **(B)** the arbuscular mycorrhizal guild specifically, across treatments and cultivars. Relative abundance was calculated from ASV-level data and expressed as the proportion of total fungal reads per sample. Differences in ASV richness for **(C)** symbiotic guilds (PERMANOVA, p = 0.001, F = 9.15) and **(D)** the arbuscular mycorrhizal guild (Wilcoxon test, p <0.05) were assessed between drought and control conditions within each cultivar and bulk soil. The difference between lowercase letters in the contrasts of each bean genotype and the soil bulk indicates a significant difference (Wilcoxon test, p <0.05).

We also identified five fungal trophic guilds across the dataset. Among ASVs assigned to symbiotic guilds, the community was predominantly composed of arbuscular mycorrhizal and ectomycorrhizal fungi, with arbuscular mycorrhizal fungi representing the dominant symbiotrophic group across all cultivars and water regimes (**Figure 4**). The relative abundance of arbuscular mycorrhizal fungi varied among cultivars and environmental contrasts, reaching the highest values in the drought-tolerant cultivar SEA5 under both well-watered and drought conditions, followed by BAT477 under drought stress. In contrast, the drought-susceptible cultivars IAC-Milênio and IAC-80SH exhibited consistently lower relative abundances of this guild, with the most pronounced reduction observed for IAC-80SH under drought conditions (**Figures 4A, B**).

Our analysis of overall guild composition revealed significant differences in the richness of symbiotrophic communities among cultivars and between water regimes (PERMANOVA, p = 0.001, F = 9.15, permutations = 999; **Figure 4C**). Patterns of ASVs richness within the arbuscular mycorrhizal guild further supported these genotype-dependent responses. Under drought stress, arbuscular mycorrhizal ASV richness was increased or maintained in the drought-tolerant cultivars BAT477 (Wilcoxon rank-sum test, p = 0.041) and SEA5 (Wilcoxon rank-sum test, p = 0.9145; **Figure 4D**), respectively. In contrast, the drought-susceptible cultivars displayed divergent responses, with IAC-Milênio showing no significant change in richness (Wilcoxon rank-sum test, p = 0.6168), whereas IAC-80SH exhibited a significant reduction in arbuscular mycorrhizal ASV richness under drought conditions (Wilcoxon rank-sum test, p = 0.0355; **Figure 4D**).

### 3.3 Association between AMF community and plant morphometric and nutritional attributes

**Figure 5:**
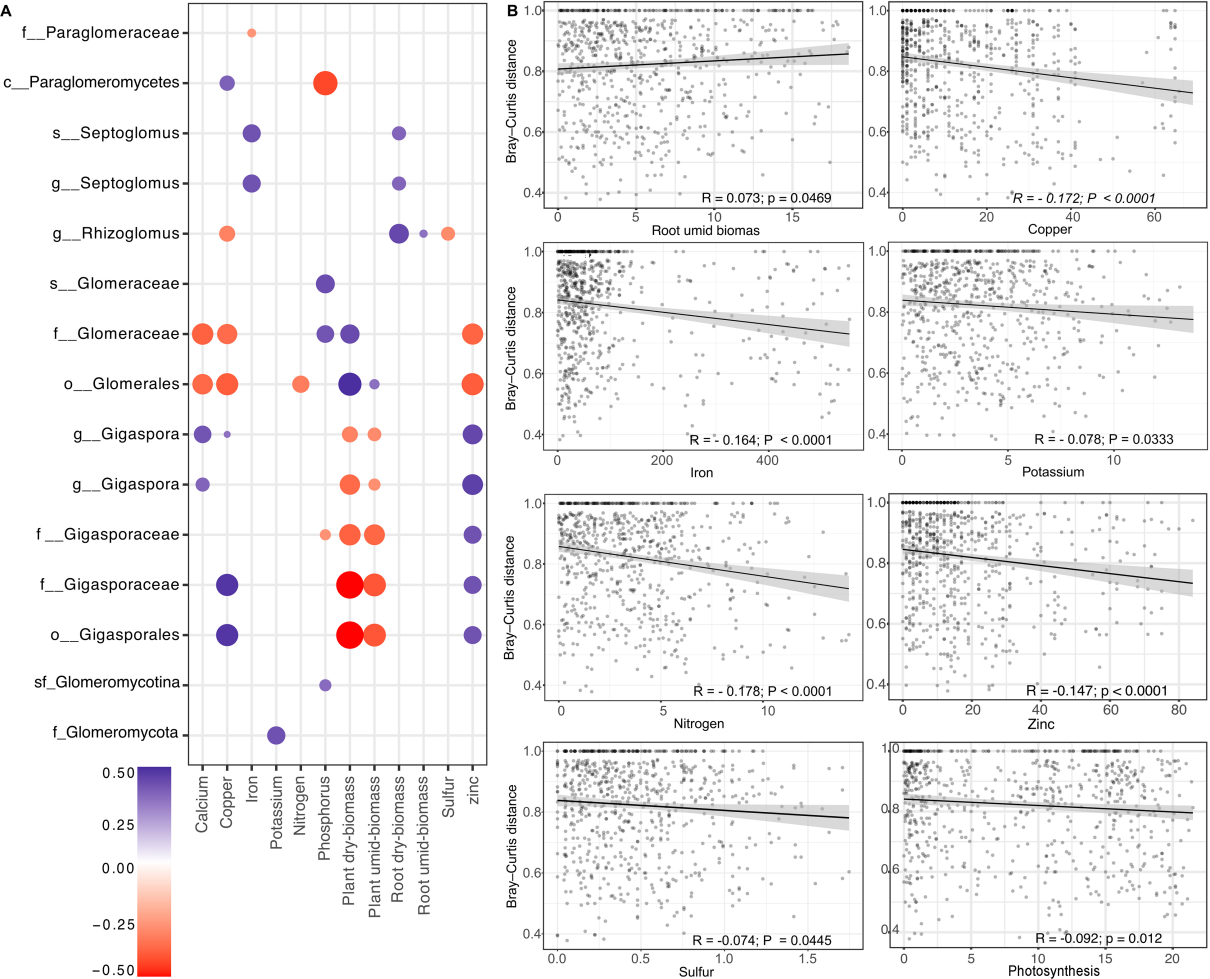
Relationships between plant nutritional and biometric parameters and arbuscular mycorrhizal fungi (AMF) abundance at the ASV level. **(A)** Correlation matrix showing significant Spearman correlations (p <0.05) between plant morphometric and nutritional variables and AMF ASV abundances. Circle color indicates the direction and magnitude of the Spearman correlation coefficient (r), while circle size reflects statistical significance. **(B)** Scatter plots showing selected significant associations from panel **(A)**, with fitted linear regression lines and 95% confidence intervals. ASVs are labeled according to the highest taxonomic resolution obtained (class, order, family, genus, or species), reflecting differences in classification resolution across sequences. All statistical analyses were conducted using ASV-level abundance data.

Through Spearman’s correlation analysis, we showed that associations between plant tissue nutrient concentrations, biometric traits, and AMF ASVs varied in both direction and strength (p <0.05; **Figure 5A**). Individual AMF ASVs displayed positive or negative correlations with multiple nutrients, including calcium, copper, iron, potassium, nitrogen, phosphorus, sulfur, and zinc. Overall, ASVs affiliated with Gigasporales were predominantly positively correlated with calcium, copper, and zinc, whereas Glomerales ASVs exhibited negative correlations with these elements. In contrast, Glomerales ASVs showed positive associations with foliar phosphorus content, while ASVs belonging to Claroideoglomerales were mainly negatively correlated with this nutrient.

Patterns involving plant biometric traits also differed among taxonomic groups. Shoot biomass was negatively correlated with the abundance of Gigasporales ASVs and positively correlated with Glomerales ASVs. Root fresh biomass, in turn, exhibited exclusively positive correlations, particularly with Glomerales ASVs such as *Septoglomus* and *Rhizoglomus*. We also demonstrated that among the biometric variables evaluated, only root fresh biomass was positively and significantly associated with AMF community structure in the rhizosphere (R = 0.073, p = 0.0469), indicating that increases in root biomass were accompanied by measurable shifts in AMF community composition (**Figure 5B**). In the same way, only photosynthesis was significantly associated with AMF community dissimilarity (R = −0.092, p <0.05) among physiological attributes. In contrast, foliar concentrations of copper, iron, potassium, nitrogen, zinc, and sulfur were negatively and significantly related to AMF community dissimilarity in the rhizosphere (p <0.05; **Figure 5B**), with the most pronounced variation observed in Fe, N, and Zn.

## 4 Discussion

### 4.1 Structural and diversity patterns of AMF communities

Our results demonstrate that the response of AMF communities to drought is substantially dependent on host genotype. While AMF community structure remained largely stable between control and drought conditions in the rhizosphere of drought-tolerant common bean genotypes, pronounced structural shifts were observed in drought-susceptible genotypes exposed to the same water deficit. This contrasting behavior indicates that drought tolerance is associated with the capacity of the host plant to maintain a structurally resilient mycorrhizal community, whereas susceptible genotypes undergo modification of the symbiosis under stress. Together, these patterns suggest that host-mediated regulation of AMF community structure represents an important component of plant adaptive responses to drought. This structural stability is consistent with previous findings in which drought-tolerant genotypes exhibited more stable physiological performance and enrichment of stress-related microbial functions under water deficit (Silva et al., 2025).

In addition to these structural shifts, differences in AMF community responses were also reflected in contrasting patterns of ASVs richness and diversity. Drought-susceptible genotypes exhibited a tendency toward reduced AMF richness and diversity under water deficit, whereas drought-tolerant genotypes maintained or even increased these attributes. These patterns are consistent with evidence suggesting that plant adaptation to extreme water limitation may partly rely on stable and functional mycorrhizal associations (Cosme, 2023). Furthermore, synergistic plant–AMF contributions to drought tolerance appear to be genotype-dependent (Rotoni et al., 2024), potentially mediated by host-controlled chemical signaling pathways involving strigolactones and lipo-chito-oligosaccharide receptors that regulate symbiotic recognition and hyphopodium formation (Araujo et al., 2025b; Haichar et al., 2014; Xu et al., 2023). Beyond early recognition, the functional establishment of arbuscules depends on species-specific host genes, such as *RAM1/2* (Reduced Arbuscular Mycorrhization 1), which control the production of cutin monomers required for successful mycorrhizal colonization (Wang et al., 2012), a mechanism consistent with the enhanced symbiotic stability observed in drought-tolerant common bean genotypes.

Despite these differences in AMF richness and diversity, no pronounced taxonomic differentiation was observed among common bean genotypes. Taxonomic assignment allowed the identification of four families and six genera among classified ASVs. At sequence-level resolution, approximately 68% of AMF ASVs were shared among genotypes under at least one environmental condition. This pattern supports the view that shared soil history constitutes a major determinant of AMF community assembly (Albracht et al., 2024), as the pool of potentially recruitable fungi was already present in the original bulk soil.

Nevertheless, despite the high degree of taxonomic overlap among genotypes, AMF community dissimilarities exceeding 90% were detected between environmental conditions within individual genotypes. Similarity percentage analysis revealed that these differences were largely driven by changes in the relative abundance of a limited subset of ASVs that contributed most strongly to community dissimilarity, with contrasting patterns of abundance maintenance and gain or loss in drought-tolerant and drought-susceptible genotypes, respectively. These results indicate that AMF community reorganization under water stress is primarily mediated by shifts in the relative contributions of the top ASVs at sequence-level resolution rather than by extensive turnover in higher taxonomic ranks. This fine-scale structural adjustment parallels previously reported functional shifts in the rhizosphere microbiome of the same genotypes under drought (Silva et al., 2025). Together, these findings suggest that structural and functional changes may occur concomitantly under water stress, although causal relationships cannot be inferred from the available data.

This differential capacity for mycorrhizal community reorganization or maintenance may reflect genotypic variation in the efficiency of conserved symbiotic signaling pathways. In particular, pathways mediated by the *CASTOR* and *POLLUX* genes, which encode calcium channels involved in Ca^2+^-dependent activation of downstream kinases and Cyclops-type transcriptional regulators, are essential for arbuscule formation and stabilization (Bonfante and Genre, 2010; Miller et al., 2014). Variation in the efficiency of these signaling components likely contributes to host-specific symbiotic preferences and to the functional maintenance of mycorrhizal associations under drought conditions (Campos et al., 2018).

In this context, more productive and drought-tolerant genotypes tend to exhibit greater nutritional homeostasis, which enables sustained root exudation and carbon allocation to fungal hyphae, key processes underlying adaptive AMF recruitment under water stress through the so-called “cry-for-help” mechanism (Bahadur et al., 2019; Boutasknit et al., 2021). By promoting the persistence of hyphal networks and spores in the soil, mycorrhizal plants can maintain improved root hydraulic conductivity, partly mediated by the regulation of aquaporin genes such as *GintAQPF1/2* and *PIP*, and experience reduced oxidative stress through enhanced activity of antioxidant enzymes, including superoxide dismutase (SOD), catalase (CAT), ascorbate peroxidase (APX), glutaredoxin (GRX), and peroxidase (POD) (Bahadur et al., 2019; Cheng et al., 2021; Li et al., 2019).

Overall, our results align with previous studies showing that host genotype influences the restructuring of AMF communities while overall taxonomic composition remains largely conserved (Marrassini et al., 2024). Because this AMF-host dialogue, mediated by strigolactones, cutin monomers, and chitin-related molecules, reflects a long history of coevolution (Bonfante and Genre, 2015), our findings support the hypothesis that drought-tolerant genotypes preserve mycorrhizal symbioses more effectively under extreme drought events. Moreover, the capacity of AMF to mitigate drought impacts in the rhizosphere appears to depend on emergent community-level properties, underscoring the need for an integrated ecological perspective to fully understand mycorrhizal symbiosis functionality (Antunes et al., 2025; Chagnon et al., 2013; Powell and Rillig, 2018).

### 4.2 Ecological strategy, trophic functions, and functional stability

CLAM analysis showed that while drought-tolerant genotypes maintained or increased the contribution of specialist ASVs under water deficit, susceptible genotypes displayed a structural redistribution toward rare amplicon variants, suggesting contrasting community assembly responses to drought among host genotypes. The host plant genotype thus functions as an ecological filter, determining whether AMF specialists adapted to stressful environments can establish and persist in the rhizosphere (Lewe et al., 2025). Importantly, the occupation of specialized niches arises from interactions between host traits and the prevailing abiotic stress regime, such as drought, rather than from any single isolated factor (Bahadur et al., 2019; Cheng et al., 2021; Mansfield et al., 2023). Within this framework, the active specialization observed in BAT477 and the functional plasticity evidenced in SEA5 represent contrasting yet equally adaptive strategies that stabilize functional niches and sustain symbiotic efficiency under water stress.

Consistent with niche occupancy patterns, FUNGuild analysis further revealed the maintenance of functional integrity within mycorrhizal symbioses in drought-tolerant genotypes, whereas the drought-susceptible genotype IAC-80SH exhibited a simultaneous reduction in relative abundance, ASV richness, and proportional representation of AMF-assigned functional guilds. These findings reinforce the notion that drought susceptibility is associated not only with losses in ASV richness but also with alteration in the contribution of functional patterns of the mycorrhizal symbiosis, thereby compromising its capacity to provide adaptive benefits to the host (Lehmann et al., 2012; Powell and Rillig, 2018; Tang et al., 2024). Together, these analyses indicate that drought tolerance in common bean is closely linked to the ability to preserve both ecological specialization and functional integrity within AMF communities. When interpreted alongside previous functional analyses of the rhizosphere microbiome in the common bean system, these results support the view that drought tolerance emerges from coordinated structural, ecological, and functional stabilization of belowground microbial communities (Silva et al., 2025).

### 4.3 Cooperation AMF-Tolerant genotypes for growth and nutrient acquisition

Our results indicate that specific groups of arbuscular mycorrhizal fungi are functionally integrated with plant nutrient acquisition and redistribution. ASVs affiliated with Gigasporales showed positive associations with foliar micronutrients and calcium, but were negatively correlated with shoot biomass. This pattern is consistent with a more specialized nutrient-acquisition strategy that may impose higher carbon costs on the host. Indeed, the competitive traits of Gigasporales, such as extensive extraradical mycelium, large spore size, and high competitiveness in nutrient-poor soils, support the interpretation that these taxa enhance nutrient uptake at the expense of increased carbon investment (Antunes et al., 2025; Chagnon et al., 2013).

In contrast, ASVs affiliated with Glomerales showed positive associations with phosphorus acquisition and with increases in both shoot and root biomass. These relationships are consistent with the ruderal life-history traits commonly attributed to this group, particularly within the genus *Glomus*, which is characterized by rapid hyphal growth, high capacity for hyphal fusion and repair, early investment in sporulation, and resilience to soil disturbance (Antunes et al., 2025; Chagnon et al., 2013). Moreover, the strong functional linkage between Glomerales and phosphorus acquisition has been consistently reported in recent studies, reinforcing their role in sustaining host growth under variable environmental conditions (Wu et al., 2024).

At the community level, the positive association between root fresh biomass and AMF community dissimilarity, although weak in magnitude, suggests that increases in root growth are accompanied by subtle structural shifts in the rhizosphere mycorrhizal assemblage. Larger root systems likely generate greater ecological space and a wider range of microhabitats and exudation patterns, thereby promoting active recruitment and turnover of AMF ASVs and reflecting a highly dynamic symbiotic interface (Lewe et al., 2025; Li et al., 2023). In contrast, the negative relationships observed between foliar nutrient concentrations (particularly nitrogen, copper, iron, and zinc) and AMF community dissimilarity indicate that plants with higher nutritional status tend to harbor comparatively more stable AMF communities. Although the explanatory power of these associations remains modest, the consistency of negative trends suggests a coordinated alignment between host nutritional condition and rhizosphere community stability, rather than a direct causal effect of AMF composition on nutrient concentration (Li et al., 2023).

In light of our results, this coupling is particularly relevant because drought-susceptible genotypes exhibit a loss of specialist AMF ASVs, leading to increased community dissimilarity and, ultimately, reduced nutritional performance. Collectively, our findings reinforce the view that plant performance under drought is linked not merely to AMF diversity per se, but to the maintenance of stable and functional mycorrhizal assemblages at sequence-level resolution. This interpretation aligns with previous findings showing that drought-tolerant bean genotypes sustain broader microbiome-level functional capacities related to nutrient cycling and stress mitigation, without implying that such functions can be attributed to AMF alone (Silva et al., 2025).

Our results are consistent with both theoretical and empirical evidence demonstrating that mycorrhizal symbioses are stabilized through reciprocal rewards and preferential allocation of resources toward beneficial partners (Bever et al., 2009; Kiers et al., 2011). Host plants are therefore not passive recipients of fungal services, but actively modulate carbon allocation and symbiotic investment in response to fungal performance, a process under genetic control that strongly influences agricultural outcomes (Ramírez-Flores et al., 2020). In this context, the ability of drought-tolerant genotypes to maintain specialized and functionally stable AMF communities likely reflects an enhanced capacity for partner selection and symbiotic regulation, thereby contributing to improved nutritional status and resilience under water-limited conditions.

## 5 Conclusion

Our results indicate that drought tolerance in common bean may be partly associated with the maintenance of stable mycorrhizal symbioses, as revealed by ASV-based community analyses. Rather than being determined solely by the presence of arbuscular mycorrhizal fungi or by higher-rank taxonomic identity, drought tolerance appears to be linked to the host plant’s capacity to structure and maintain AMF communities under water stress. In this context, the contribution of AMF to plant performance may depend on the ability of drought-tolerant genotypes to promote adaptive strategies of fungal community specialization or functional plasticity. Within a system of reciprocal rewards, the structural stability of plant–AMF interactions may contribute to improved nutrient acquisition, growth, and resilience under water-limited conditions. Accordingly, our findings highlight the importance of identifying host traits that support the functional and structural stability of mycorrhizal symbioses within the plant microbiome at a sequence-resolved community level. By framing drought tolerance as a property potentially influenced by host–microbiome interactions, this study provides a conceptual basis for future research exploring whether mycorrhizal functionality could be considered in strategies aimed at developing crops resilient to increasing climatic variability.

## Data Availability

The datasets presented in this study can be found in online repositories. The names of the repository/repositories and accession number(s) can be found in the article/**Supplementary Material**.
