# Supplementary material for: Genotype-dependent stability and specialization of arbuscular mycorrhizal fungal communities under drought in common bean
Source: Front Plant Sci. 2026 May 1;17:1786322. doi: 10.3389/fpls.2026.1786322 (PMC13176285; doi:10.3389/fpls.2026.1786322)
Supplement: Supplementary file 1 [file DataSheet1.pdf]

Supplementary Material

GENOTYPE-DEPENDENT STABILITY AND SPECIALIZATION OF ARBUSCULAR MYCORRHIZAL FUNGAL COMMUNITIES UNDER DROUGHT IN COMMON BEAN

Maycon Cristiano Barbosa[1], Thierry Alexandre Pellegrinetti[1], Izadora de Cássia Mesquita da Cunha[1], Ana Vitória Reina da Silva[1], Eduardo Henrique Marcandalli Boleta[1], Lara de Almeida Losovoi[1], Rodrigo Mendes[2], Siu Mui Tsai[1], Lucas William Mendes[1]

[1] Center for Nuclear Energy in Agriculture, University of São Paulo, Piracicaba, SP, Brazil

[2] Brazilian Agricultural Research Corporation, Embrapa Environment, Jaguariuna, Brazil.

Corresponding author: lwmendes@cena.usp.br

Supplementary Tables: S1 to S5

Supplementary Figure: S1 to S2

Suppl Table 1. Plant morphometric and physiological attributes of different common bean cultivars (BAT477, SEA5, M, 80SH) during drought stress (W-) and well watered (W+) conditions.

**Plant Genotype**	**High**	**Root UB**	**Root DB**	**Plant UB**	**Plant DB**	**Photosyntesis**	**Stoma conduct**	**CO2 inside cell**	**Transpiration**
	cm	Weight (g/plant)				umol CO2/m2/s	mol H2O/m2/s	umol CO2/mol seiva	mmol H2O/m2/s
Bulk Soil	-	-	-	-	-	-	-	-	-
BAT477 W+	125.2± 19.61	17.48± 6.25	9.63± 4.45	149.4+1 1.96	24.42+ 2.26	15.64± 2.80	0.416± 0.08	296.36± 6.85	7.52± 0.99
BAT477 W-	111.4± 30.23	8.724± 2.08	4.19± 1.03	77.86± 24.59	15.78± 3.26	2.68± 0.62	0.022± 0.004	151.56± 54.74	0.46± 0.21
Sea5 W+	69.4± 7.44	11.24± 4.36	5.24± 2.09	86.54± 27.91	13.91± 5.12	17.12± 4.12	0.344± 0.16	268.34± 18.34	6.28± 2.26
Sea5 W-	68.0± 11.59	5.36±1. 29	2.43± 0.75	58.17± 6.95	12.41± 2.55	2.24± 0.63	0.03± 0.01	210.54± 86.5	0.68± 0.25
IAC-80SH W+	132± 17.9	12.51± 3.13	6.9± 1.83	122.72± 41.45	21.29± 7.86	18.9± 3.39	0.436±0.13	281.58± 15.93	7.74± 1.89
IAC-80SH W-	110± 27.61	9.71± 3.60	5.486±2 .86	93.7±16 .72	19.344± 3.01	2.32± 0.55	0.036± 0.04	169.48± 114.62	0.62± 0.42
IAC-Milenio W+	129.2± 31.94	14.21± 5.24	6.88± 3.57	156.12± 34.41	22.98± 8.12	16.66± 2.34	0.32± 0.07	272.38± 7.74	6.06± 1.11
IAC-Milenio W-	100.8± 37.29	9.42± 3.22	5.26± 2.25	86.24± 5.89	15.37± 2.18	2.08± 0.87	0.024± 0.011	234.08± 61.34	0.5± 0.35

UB = fresh biomass; DB = dry biomass

Suppl Table 2. Shoot nutritional parameters of different common bean cultivars (BAT477, SEA5, M, 80SH) during drought stress (W-) and well watered (W+) conditions.

Plant Genotype	N	P	K	Ca	Mg	S	B	Cu	Fe	Mn	Zn
Bulk Soil	-	-	-	-	-	-	-	-	-		
BAT477 W+	26.35± 0.66	2.98± 0.32	22.24± 1.78	10.86± 1.32	4.23± 0.31	2.19± 0.40	64.02± 10.59	10.8± 4.08	191.4± 44.64	891.8± 90.13	51.0± 4.30
BAT477 W-	27.18± 4.89	3.19± 0.13	27.79± 1.63	11.43± 2.79	4.02± 0.67	2.25± 0.28	52.03± 4,45	19.40± 17.09	138.6± 43.07	1000± 405.03	51.4± 6.69
Sea5 W+	28.056± 4.26	2.91± 0.12	24.97± 3.41	12.39± 2.17	4.10± 0.44	1.99± 0.19	64.38± 14.87	16.2± 5.76	124.2± 21.61	878.8± 304.32	61.2± 9.28
Sea5 W-	24.92± 2.40	2.62± 0.15	26.72± 3.65	15.24± 1.78	4.35± 0.19	2.46± 0.42	62.22± 7.31	42.4± 20.83	367.6± 235.51	1323.4± 464.87	92.2±19. 94
IAC-80SH W+	25.98± 3.93	5.04± 2.27	23.53± 3.35	9.53± 1.36	4.255± 0.42	1.58± 0.24	59.85± 10.35	17.8± 13.88	181.6± 35.16	1073.8± 528.75	56.6± 11.30
IAC-80SH W-	24.04± 2.07	5.37± 3.35	25.34± 1.62	7.76± 1.75	3.82± 0.55	1.49± 0.11	64.25± 7.91	8.20± 0.83	136.8± 25.25	919± 375.16	46.8± 6.41
IAC-Milenio W+	25.15± 2.14	2.95± 0.08	23.05± 1.95	9.62± 2.48	3.57± 0.41	1.98± 0.38	58.91± 7.52	8.60± 1.34	138± 49,89	702.4± 337.86	50.4± 9.18
IAC-Milenio W-	26.698± 3.62	3.16± 0.38	26.57± 3.88	10.57± 1.78	3.76± 0.50	1.91± 0.34	68.30± 6.88	22.2± 12.01	157.4± 18.55	968± 310.05	44.0± 9.64

N = Nitogen; P = Phosphorus; K = Potassium; Ca = Calcium, = Mg = Magnesium; S = súlfur; B = Boron; Cu = cupper; Fe = Iron; Mn = manganese; Zn = Zinc

Suppl Table 3. Soil chemical parameters of the bulk and rhizosphere soils of four common bean cultivars during drought stress (W-) and well watered (W+).

Plant Genotype	pH	OM	P	K	Ca	Mg	H+Al	Al	So.B	CTC	Sa.B	S.Al	S	Cu	Fe	Zn	Mn	B
Bulk Soil W+	4.4± 0.01	9.8± 1.1	147± 40.9	7.94 ±0.8	17.4 ±2.6	11.4 ±1.9	18.8 ±2.3	1.4± 0.5	36.8 ±4.7	55.6 ± 5.3	66± 3.7	3.6± 1.3	49± 14.7	19.6 ±10.6	37. ±3.1	4.6± 0.9	38.8 ± 5.4	1.1± 0.2
Bulk Soil W-	4.7± 0.2	10.4 ± 2.2	243± 28.5	18.5 ± 6.1	31.2 ± 5.9	26.2 ± 4.8	17.8 ± 3	0.8± 0.4	75.8 ± 15.9	93.6 ± 15.1	80.4 ± 4.8	1.2± 0.8	111± 14.7	4.2± 6.5	32.4 ± 4.8	8.8± 1.4	68.6 ± 17.3	1.8± 0.3
BAT477 W+	4.2± 0.12	9±0.0	84.6± 20.1	1.74± 0.19	6.4± 1.14	4.8± 0.83	16.8± 1.09	2.2± 0.44	13.2± 1.92	30± 2.54	43.4± 3.78	14.8± 2.58	8.2± 6.64	1.37± 0.24	40.24 ± 4.35	2.916 ± 0.68	14.48 ± 2.61	0.592 ± 0.14
BAT477 W-	4.46± 0.11	10.6± 0.89	114± 7.03	2.74± 0.43	10.2± 1.64	7.8± 0.83	19± 3.46	1.2± 0.44	20.8± 2.16	39.8± 2.16	52.4± 6.46	5.4± 2.60	34± 2.12	1.42± 0.06	38.96 ± 4.45	3.268 ± 0.44	21.52 ± 3.39	0.73± 0.05
Sea5 W+	4.3± 0.12	9.8± 1.09	102± 16.9	2.18± 0.57	8± 2.0	6.2± 1.64	17.2± 1.79	2±0	16.6± 4.09	33.8± 4.14	48.2± 7.01	11.6± 2.5	15.6± 13.2	1.52± 0.37	38.32 ± 2.77	3.476 ± 0.38	18.48 ± 5.20	0.566 ± 0.11
Sea5 W-	4.58± 0.08	10.2± 1.09	114± 19.8	3.1± 0.78	11.6± 1.14	8.4± 1.14	17± 2.0	1± 0	23.2± 1.16	40.2± 3.76	57.8± 1.82	4.4± 0.54	33.4± 2.40	1.328 ± 0.08	38.4± 1.71	3.50± 0.55	22.64 ± 3.08	0.87± 0.26
IAC-80SH W+	4.25± 0.1	9.5± 1.0	107.3 ± 14.9	1.75± 0.26	7.5± 1.29	5.5± 0.57	16.75 ± 2.21	2±0.0	15± 1.15	31.75 ± 1.71	46.75 ± 4.79	11.75 ± 0.95	20.5± 5.91	1.2± 0.11	38.8± 3.09	2.835 ± 0.22	18.6± 3.40	0.60± 0.07
IAC-80SH W-	4.46 ± 0.11	11.2± 1.78	102± 34.4	2.38± 0.44	10.4± 2.19	8± 2.23	16.2± 1.09	1.2± 0.44	20.8± 4.76	37± 4.94	55.6± 5.98	5.8± 3.49	36.8± 3.03	1.344 ± 0.15	39.68 ± 5.72	3.228 ± 0.55	20.96 ± 3.69	0.63± 0.09
IAC-Milenio W+	4.3± 0.07	9.4± 0.89	84.6± 24.6	1.84± 0.59	7± 0.7	5.4± 0.89	17.6± 2.19	2.2± 0.44	14.4± 2.07	32± 2.34	44.6± 5.94	13.6± 2.88	11.4± 9.23	1.344 ± 0.15	39.12 ± 3.25	3.088 ± 0.53	15.2± 3.86	0.59± 0.11

IAC-Milenio W-	4.46± 0.05	10.2± 1.09	101.4 ± 21.0	2.72± 0.37	10.6± 1.81	7.4± 2.50	17± 1.41	1.2± 0.44	20.8± 3.83	37.8± 4.49	54.6± 5.02	5.4± 1.94	27.2± 11.5	1.38± 0.09	40± 2.46	3.04± 0.21	20± 1.67	0.76± 0.08

OM = Organic matter; P = Phosphorus; K = Potassium; Ca = Calcium; Mg = Magnesium; H+Al = Potential Acidity; CTC = Cation change capacity; SB = Sum bases ; S = Sulfur; Fe = Iron; Zn = Zinc; Mn = Manganese; B = Borun

Suppl Table 4. Fitting environmental variables to a PCoA ordination plot with respectives determination coefficients (R2) and significance (Pr>(r)).

Plant Genotype	Soil Chemical Attribute	Dim1	Dim2	R2	Pr(>r)
BAT477	pH	-0.08135	-0.99669	0.2610	0.379
	Organic Matter	-0.29093	-0.95675	0.4342	0.150
	Phosphorus	0.32772	-0.94478	0.3106	0.303
Sea5	pH	-0.77385	0.63336	0.1066	0.693
	OM	-0.17377	0.98479	0.0019	0.992
	P	0.24288	-0.97006	0.0733	0.756
IAC-Milenio	pH	-0.88297	0.46944	0.5977	0.037
	OM	-0.75184	0.65935	0.3971	0.168
	P	-0.87632	0.48173	0.225	0.419
IAC-80SH	pH	0.71232	-0.70186	0.1215	0.368
	OM	0.97586	-0.21841	0.2617	0.337
	P	0.64623	0..76314	0.0215	0.904

Sppl Table 5. Result of multinomial species classification method (CLAM) for the comparison between drought and control condition for each bean genotype

Bean	ASVs	Total _no_stress	Total_stressed	Classes
BAT	M_asv_2126	2	5	Too_rare
BAT	M_asv_4159	22	15	Generalist
BAT	M_ asv _5202	1	1	Too_rare
BAT	M_ asv _5599	1	9	Too_rare
BAT	M_ asv _5661	3	11	Generalist
BAT	M_ asv _5729	1	9	Too_rare
BAT	M_ asv _5753	4	0	Too_rare
BAT	M_ asv _5788	0	2	Too_rare
BAT	M_ asv _5792	0	2	Too_rare
BAT	M_ asv _5808	0	7	Too_rare
BAT	M_ asv _5812	0	3	Too_rare
BAT	M_ asv _5849	3	3	Too_rare
BAT	M_ asv _5852	1	0	Too_rare
BAT	M_ asv _5865	3	4	Too_rare
BAT	M_ asv _5995	0	1	Too_rare
BAT	M_ asv _6108	2	1	Too_rare
BAT	M_ asv _6162	4	0	Too_rare
BAT	M_ asv _6258	1	11	Specialist_BAT_stressed
BAT	M_ asv _6311	1	2	Too_rare
BAT	M_ asv _6312	2	9	Generalist
BAT	M_ asv _6320	2	0	Too_rare
BAT	M_ asv _6351	0	9	Too_rare
BAT	M_ asv _6396	0	3	Too_rare
BAT	M_ asv _6397	1	4	Too_rare

BAT	M_ asv _6416	2	0	Too_rare
BAT	M_ asv _6421	1	0	Too_rare
BAT	M_ asv _6430	0	1	Too_rare
BAT	M_ asv _6545	0	1	Too_rare
BAT	M_ asv _6546	0	2	Too_rare
BAT	M_ asv _6548	0	7	Too_rare
BAT	M_ asv _6593	0	1	Too_rare
BAT	M_ asv _6616	0	1	Too_rare
BAT	M_ asv _6628	0	1	Too_rare
BAT	M_ asv _6634	0	1	Too_rare
BAT	M_ asv _6635	1	17	Specialist_BAT_stressed
BAT	M_ asv _6638	0	8	Too_rare
BAT	M_ asv _6661	1	2	Too_rare
BAT	M_ asv _6665	0	2	Too_rare
SEA	M_ asv _1386	1	0	Too_rare
SEA	M_ asv _2126	3	7	Generalist
SEA	M_ asv _4159	7	9	Generalist
SEA	M_ asv _5202	8	8	Generalist
SEA	M_ asv _5599	4	0	Too_rare
SEA	M_ asv _5661	11	13	Generalist
SEA	M_ asv _5721	0	1	Too_rare
SEA	M_ asv _5729	11	6	Generalist
SEA	M_ asv _5788	1	0	Too_rare
SEA	M_ asv _5812	5	1	Too_rare
SEA	M_ asv _5849	1	2	Too_rare
SEA	M_ asv _5861	0	1	Too_rare
SEA	M_ asv _5915	1	0	Too_rare
SEA	M_ asv _5995	2	1	Too_rare
SEA	M_ asv _6312	1	1	Too_rare
SEA	M_ asv _6320	0	2	Too_rare
SEA	M_ asv _6545	6	3	Generalist
SEA	M_ asv _6546	10	2	Specialist_SEA_no_stress
SEA	M_ asv _6548	31	11	Specialist_SEA_no_stress
SEA	M_ asv _6593	6	9	Generalist
SEA	M_ asv _6616	0	1	Too_rare
SEA	M_ asv _6628	3	2	Too_rare
SEA	M_ asv _6629	4	2	Too_rare
SEA	M_ asv _6634	4	7	Generalist
SEA	M_ asv _6635	32	41	Generalist
SEA	M_ asv _6638	5	9	Generalist
SEA	M_ asv _6661	6	10	Generalist
IAC-MIlenio	M_ asv _2126	0	4	Too_rare
IAC-MIlenio	M_ asv _4159	7	0	Too_rare
IAC-MIlenio	M_ asv _5202	3	1	Too_rare
IAC-MIlenio	M_ asv _5599	0	2	Too_rare

IAC-MIlenio	M_ asv _5661	7	0	Too_rare
IAC-MIlenio	M_ asv _5704	1	0	Too_rare
IAC-MIlenio	M_ asv _5721	4	0	Too_rare
IAC-MIlenio	M_ asv _5729	4	0	Too_rare
IAC-MIlenio	M_ asv _5753	2	0	Too_rare
IAC-MIlenio	M_ asv _5792	0	1	Too_rare
IAC-MIlenio	M_ asv _5861	9	0	Specialist_M_no_stress
IAC-MIlenio	M_ asv _5995	1	0	Too_rare
IAC-MIlenio	M_ asv _6311	0	1	Too_rare
IAC-MIlenio	M_ asv _6312	2	0	Too_rare
IAC-MIlenio	M_ asv _6323	0	1	Too_rare
IAC-MIlenio	M_ asv _6351	4	0	Too_rare
IAC-MIlenio	M_ asv _6545	3	4	Too_rare
IAC-MIlenio	M_ asv _6546	3	0	Too_rare
IAC-MIlenio	M_ asv _6593	2	0	Too_rare
IAC-MIlenio	M_ asv _6616	4	0	Too_rare
IAC-MIlenio	M_ asv _6628	4	0	Too_rare
IAC-MIlenio	M_ asv _6634	0	1	Too_rare
IAC-MIlenio	M_ asv _6635	11	4	Generalist
IAC-MIlenio	M_ asv _6638	6	4	Generalist
IAC-MIlenio	M_ asv _6661	1	0	Too_rare
IAC-MIlenio	M_ asv _6665	1	3	Too_rare
IAC-80SH	M_ asv _2126	2	1	Too_rare
IAC-80SH	M_ asv _4159	17	5	Specialist_SH_no_stress
IAC-80SH	M_ asv _5202	0	4	Too_rare
IAC-80SH	M_ asv _5599	2	0	Too_rare
IAC-80SH	M_ asv _5661	25	3	Specialist_SH_no_stress
IAC-80SH	M_ asv _5704	3	0	Too_rare
IAC-80SH	M_ asv _5721	3	0	Too_rare
IAC-80SH	M_ asv _5729	10	5	Generalist
IAC-80SH	M_ asv _5753	1	0	Too_rare
IAC-80SH	M_ asv _5788	4	0	Too_rare
IAC-80SH	M_ asv _5792	1	1	Too_rare
IAC-80SH	M_ asv _5794	0	2	Too_rare
IAC-80SH	M_ asv _5808	7	1	Specialist_SH_no_stress
IAC-80SH	M_ asv _5812	0	1	Too_rare
IAC-80SH	M_ asv _5849	3	1	Too_rare
IAC-80SH	M_ asv _5852	3	1	Too_rare
IAC-80SH	M_ asv _5995	1	2	Too_rare
IAC-80SH	M_ asv _6108	10	6	Generalist
IAC-80SH	M_ asv _6162	3	1	Too_rare
IAC-80SH	M_ asv _6166	1	0	Too_rare
IAC-80SH	M_ asv _6258	5	2	Too_rare
IAC-80SH	M_ asv _6301	2	0	Too_rare
IAC-80SH	M_ asv _6311	1	2	Too_rare

IAC-80SH	M_ asv _6312	19	4	Specialist_SH_no_stress
IAC-80SH	M_ asv _6320	8	0	Specialist_SH_no_stress
IAC-80SH	M_ asv _6321	6	0	Too_rare
IAC-80SH	M_ asv _6323	2	0	Too_rare
IAC-80SH	M_ asv _6366	3	0	Too_rare
IAC-80SH	M_ asv _6375	5	1	Too_rare
IAC-80SH	M_ asv _6382	0	4	Too_rare
IAC-80SH	M_ asv _6396	1	1	Too_rare
IAC-80SH	M_ asv _6397	1	1	Too_rare
IAC-80SH	M_ asv _6416	7	2	Generalist
IAC-80SH	M_ asv _6421	6	5	Generalist
IAC-80SH	M_ asv _6424	1	0	Too_rare
IAC-80SH	M_ asv _6430	7	0	Too_rare
IAC-80SH	M_ asv _6545	10	1	Specialist_SH_no_stress
IAC-80SH	M_ asv _6546	4	3	Too_rare
IAC-80SH	M_ asv _6548	4	1	Too_rare
IAC-80SH	M_ asv _6593	3	1	Too_rare
IAC-80SH	M_ asv _6628	1	0	Too_rare
IAC-80SH	M_ asv _6629	2	0	Too_rare
IAC-80SH	M_ asv _6634	10	1	Specialist_SH_no_stress
IAC-80SH	M_ asv _6635	40	6	Specialist_SH_no_stress
IAC-80SH	M_ asv _6638	9	0	Specialist_SH_no_stress
IAC-80SH	M_ asv _6661	9	1	Specialist_SH_no_stress
IAC-80SH	M_ asv _6665	10	0	Specialist_SH_no_stress

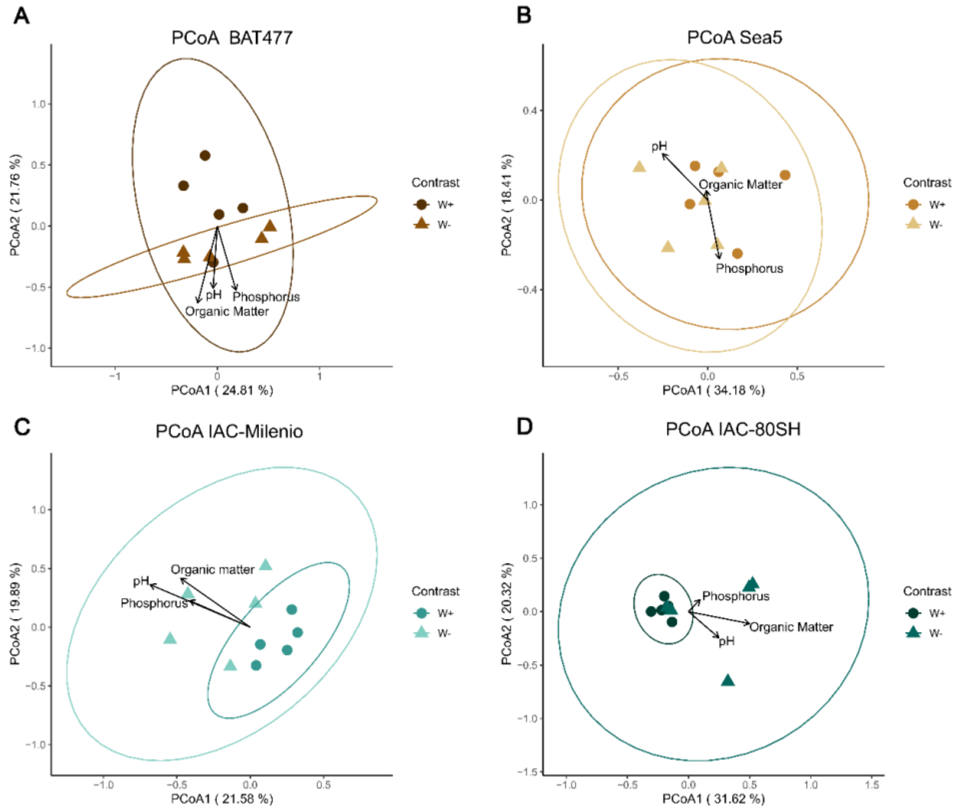

Suppl Figure S1. Principal Coordinates Analysis (PCoA) based on Bray–Curtis dissimilarities of arbuscular mycorrhizal fungal (AMF) communities in the rhizosphere of four common bean cultivars (BAT477, SEA5, IAC-Milênio and IAC-80SH), analyzed separately for each genotype under control and drought conditions. Ordinations were performed using log(1 + x)-transformed ASV abundance data. Soil chemical variables (pH, organic matter, and available phosphorus) were fitted onto each ordination using the *envfit* procedure, with vectors indicating the direction and strength of correlations with the ordination axes. The goodness of fit (R^2) and significance levels were assessed by permutation tests (999 permutations). Among the tested variables, soil pH showed a significant association with AMF community structure only for the cultivar IAC-Milênio ($p < 0.05$), whereas no significant relationships were detected for the remaining cultivars, indicating a genotype-dependent influence of soil chemical attributes on AMF community organization.

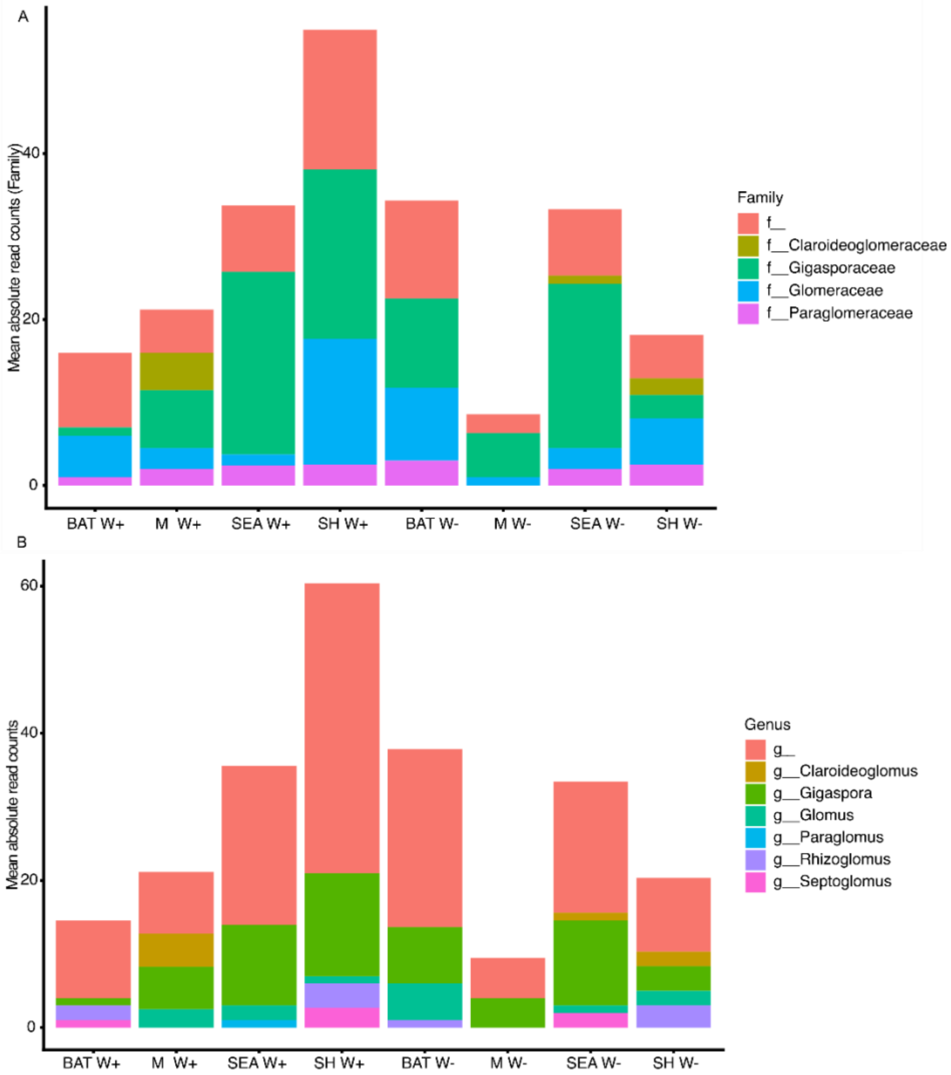

Supplementary Fig. S2. Absolute number of sequencing reads assigned to arbuscular mycorrhizal fungal (AMF) taxa in the rhizosphere microbiome of common bean cultivars under contrasting water regimes. Bar charts show the total number of reads attributed to each family (A) and genus (B) across all samples. This figure complements Fig. 2A–B by presenting the absolute counts underlying the relative abundance patterns shown in the main text.
